# Supplementary material for: Effects of Matched and Mismatched Visual Flow and Gait Speeds on Human Electrocortical Spectral Power
Source: Brain Sci. 2025 May 21;15(5):531. doi: 10.3390/brainsci15050531 (PMC12109666; doi:10.3390/brainsci15050531)
Supplement: Supplementary file 1 [file brainsci-15-00531-s001.zip › brainsci-3653162-supplementary.pdf]

Supplementary Materials:

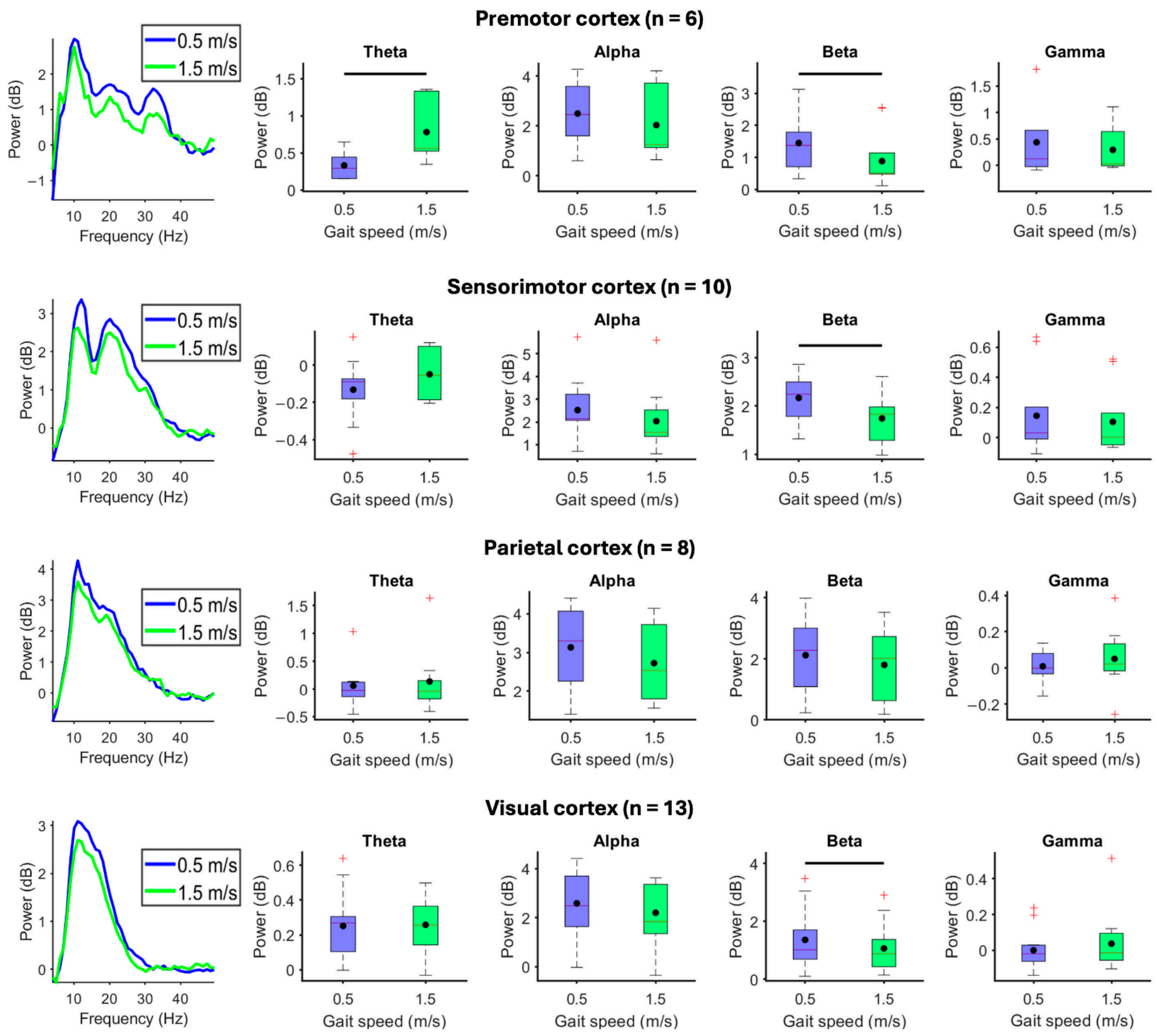

Figure S1. Effect of gait speed without visual flow. (**Left**) Mean spectral power in each gait speed condition. (**Right**) Mean spectral power boxplots aggregated within theta (4-8 Hz), alpha (8-13 Hz), beta (13-30 Hz), and gamma (30-50 Hz) bands when participants viewed a stationary virtual reality environment and walked at 0.5 or 1.5 m/s. Group means are black dots. Significant condition comparisons (black horizontal line, Wilcoxon signed rank test, $p < 0.05$). Red plus signs are outliers.

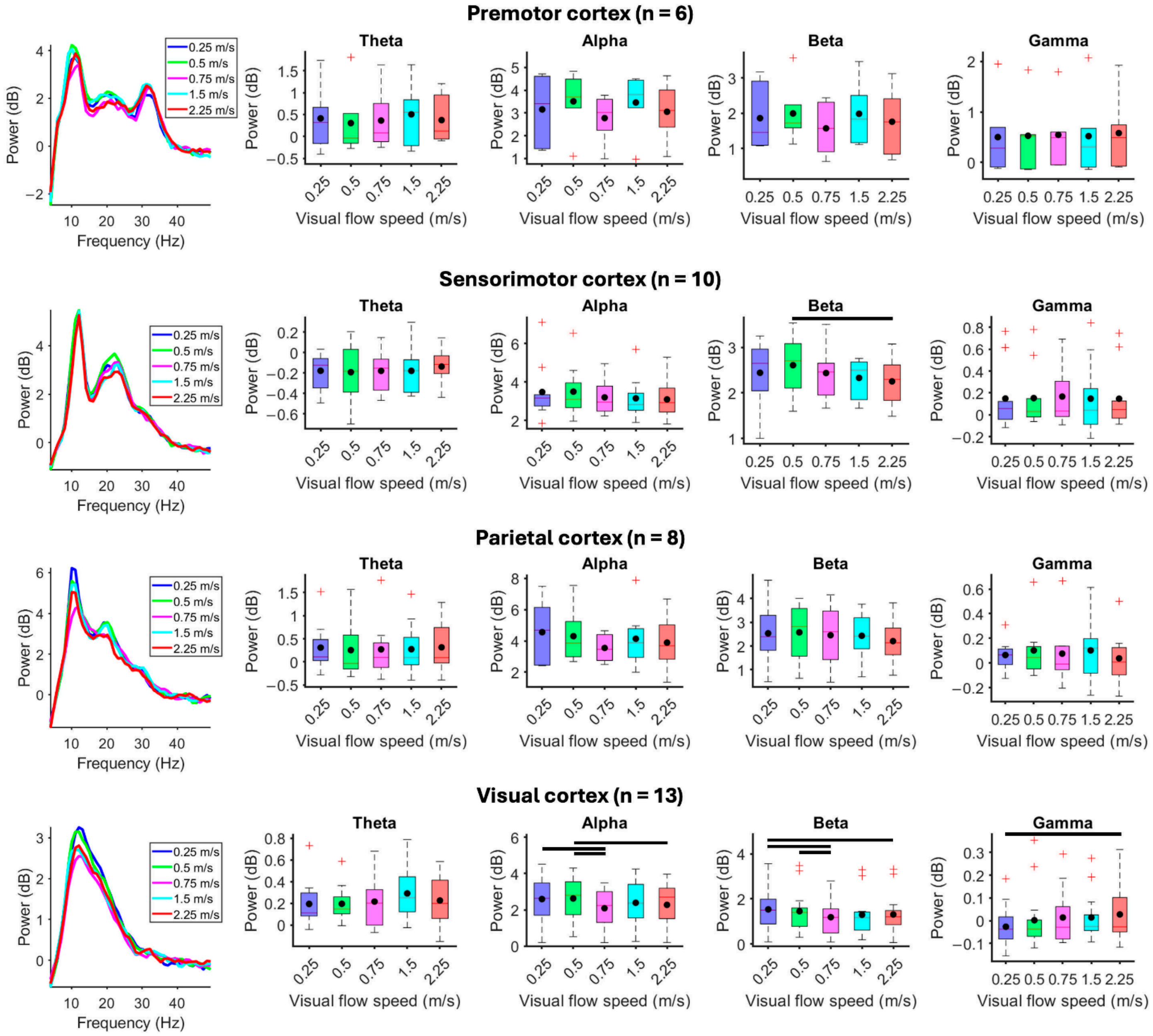

Figure S2. Effect of visual flow without walking. Subjects stood motionless on the treadmill and passively viewed the virtual reality environment moving at 0.25, 0.5, 0.75, 1.5, or 2.25 m/s. (**Left**) Mean spectral power during each visual flow speed condition. (**Right**) Mean spectral power boxplots aggregated within theta (4-8 Hz), alpha (8-13 Hz), beta (13-30 Hz), and gamma (30-50 Hz) bands. Group means are black dots. Significant condition comparisons (black horizontal lines, Wilcoxon signed rank test, $p<0.005$). Red plus signs are outliers.

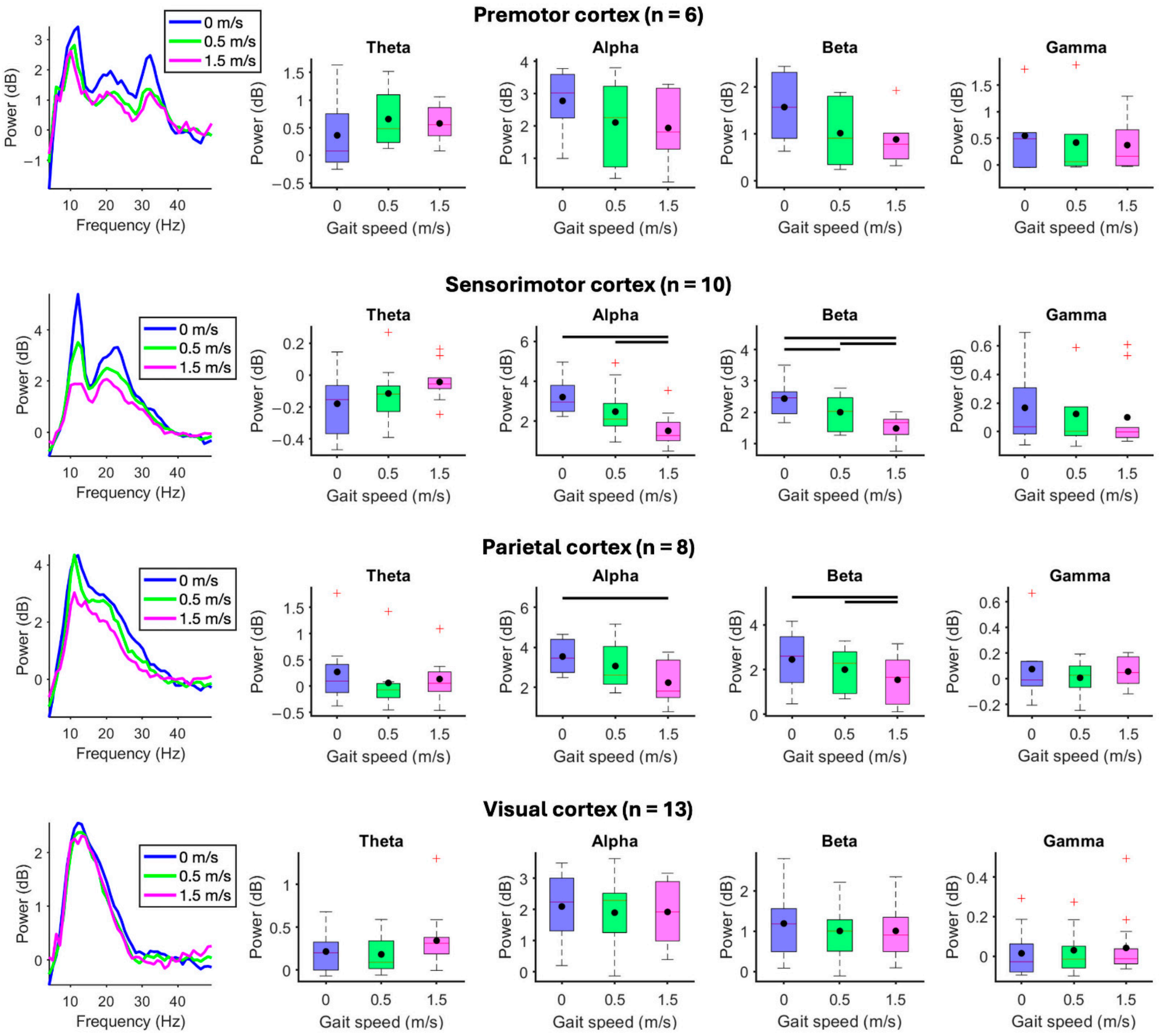

Figure S3. Effect of gait speed at fixed visual flow speed. Subjects viewed a virtual reality environment moving at 0.75 m/s while standing motionless (0 m/s) or walking at 0.5 or 1.5 m/s. (**Left**) Mean spectral power in each gait speed condition. (**Right**) Mean spectral power boxplots aggregated within theta (4-8 Hz), alpha (8-13 Hz), beta (13-30 Hz), and gamma (30-50 Hz) bands. Group means are black dots. Significant condition comparison (black horizontal line, Wilcoxon signed rank test, $p<0.017$). Red plus signs are outliers.

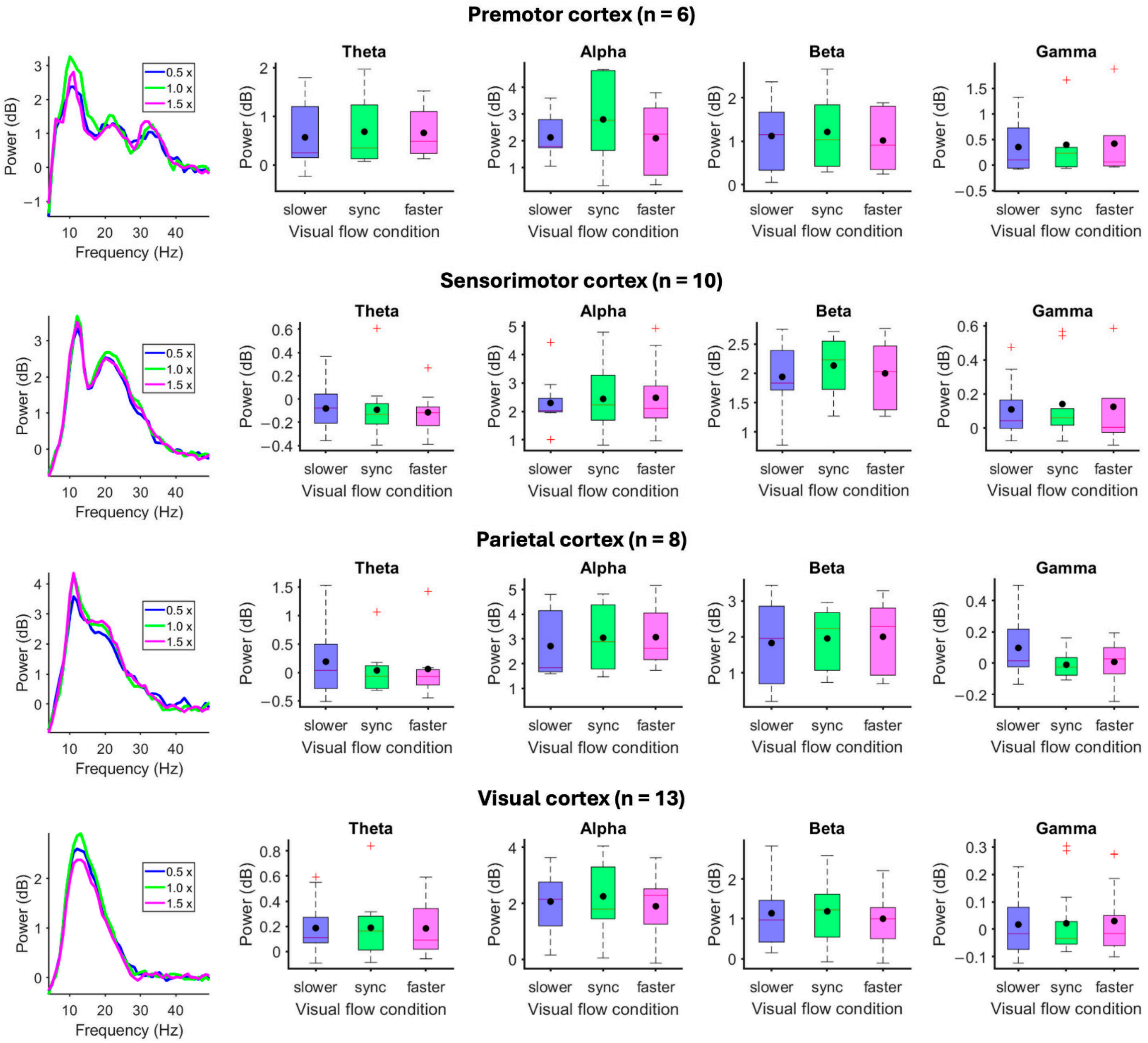

Figure S4. Effects of mismatched gait and visual flow speeds during 0.5 m/s treadmill walking. The virtual reality environment moved at 0.5x, 1x, or 1.5x gait speed. (**Left**) Mean spectral power during mismatched visual flow and gait speed. (**Right**) Mean spectral power boxplots aggregated within theta (4-8 Hz), alpha (8-13 Hz), beta (13-30 Hz), and gamma (30-50 Hz) bands. Group means are black dots. Significant condition comparisons (black horizontal line, Wilcoxon signed rank test, $p < 0.017$). Red plus signs are outliers.

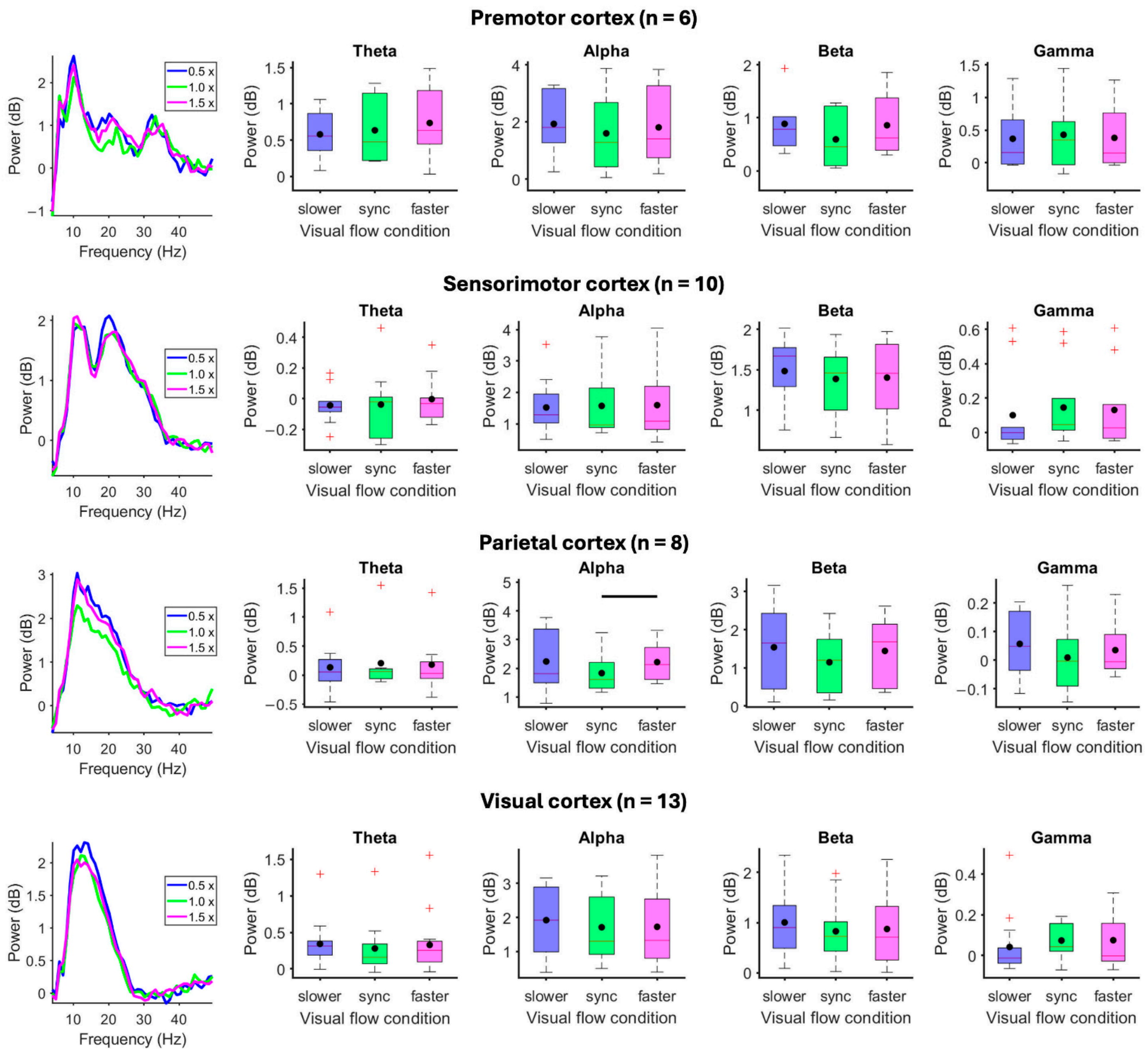

Figure S5. Effects of mismatched gait and visual flow speeds during 1.5 m/s treadmill walking. The virtual reality environment moved at 0.5x, 1x, or 1.5x gait speed. (**Left**) Mean spectral power during mismatched visual flow and gait speed. (**Right**) Mean spectral power boxplots aggregated within theta (4-8 Hz), alpha (8-13 Hz), beta (13-30 Hz), and gamma (30-50 Hz) bands. Group means are black dots. Significant condition comparisons (black horizontal line, Wilcoxon signed rank test, $p < 0.017$). Red plus signs are outliers.
